# Impact of Crop Sequence and Fertilization on Potato Yield in a Long-Term Study

**DOI:** 10.3390/plants12030495

**Published:** 2023-01-21

**Authors:** Andrzej Blecharczyk, Przemysław Łukasz Kowalczewski, Zuzanna Sawinska, Piotr Rybacki, Dominika Radzikowska-Kujawska

**Affiliations:** 1 Department of Agronomy, Poznań University of Life Sciences, 11 Dojazd St., 60-632 Poznań, Poland; 2 Department of Food Technology of Plant Origin, Poznań University of Life Sciences, 31 Wojska Polskiego St., 60-624 Poznań, Poland

**Keywords:** crop rotation, monoculture, organic and mineral fertilization, chemical composition of tubers, *Solanum tuberosum* L.

## Abstract

The research was conducted during the years 2007–2013, on the base of a long-term study established in 1958, at the Experimental Station Brody (52°26′ N; 16°18′ E), belonging to the Poznań University of Life Sciences. Varieties of potatoes resistant to cyst nematodes were grown in a seven-course crop rotation (potato—spring barley—alfalfa—alfalfa—spring oilseed rape—winter wheat—winter rye) and in continuous monoculture. The presented study from the years 2007–2013 covers the next 8th rotation of the 7-field crop rotation (since 1958). With regard to continuous cultivation, this is the period between the 50th and 56th year of the potato monoculture. The experiment included 11 fertilization variants, of which the following 7 were included in the study: 1—control object without fertilization, 2—manure, 3—manure + NPK, 4—NPKCa, 5—NPK, 6—NP, 7—NK and 8—PK. Every year, mineral and organic fertilization was applied in the following doses per 1 ha: N—90 kg, P—26 kg, K—100 kg, manure—30 t and Ca—0.7 t. Potato cultivation in monoculture resulted in a significant reduction in tuber yield compared to crop rotation and a reduction in the number of tubers per plant and the average weight of one tuber. Manure fertilization, especially in combination with NPK mineral fertilizer, had a more favorable effect on the level of potato yielding and the content of N, P, K and Mg in tubers compared to only mineral fertilization, but decreased the content of dry matter, starch and Ca. The results of long-term experiment indicate that the most effective in potato cultivation is the combined application of both manure and full mineral fertilization (NPK) with the proper sequence of plants (crop rotation).

## 1. Introduction

Potatoes (*Solanum tuberosum* L.) are one of the most important crops intended for food and feed purposes. Easy cultivation and reasonable climate requirements allow it to be grown all over the world. The plant, in its root system, produces tubers rich in starch, which is why often high-starch varieties are used in the starch process, which results in pure potato starch [1]. The first mentions of potato cultivation date to 8000 years ago and today, more than 5000 varieties of this plant are cultivated [2]. The dry mass of potato tubers reaches 20–25% and their chemical composition, determining the nutritional value, depends not only on the variety, but also on the cultivation and harvesting conditions [3]. The literature sources indicate a significant impact of storage conditions on the content of dry matter of potato tubers, content of reducing sugars or amino acid composition [4,5]. Among the nutrients forming the dry mass of potato tubers, starch, protein, vitamins, minerals and fiber should be distinguished. Potato tubers contain, among others, vitamins C, PP, B1, B2 and B6, iodine, calcium, chlorine and sulfur. Consumption of one medium-sized potato on skin (approx. 150 g) provides the recommended daily dose of vitamin C for an adult (100 mg) [6,7]. It is worth adding that potatoes are vegetables with a high iron content [8,9]. Potatoes are a low-fat raw material (about 0.5% of fresh weight), containing mainly linoleic (omega-6) and linolenic (omega-3) acids [10].

The starch content, the most valuable from a nutritional point of view, depends more on the variety than the applied agrotechnical treatments, while potato yield is mainly determined by agrotechnical factors, and less so by the choice of variety. Agrotechnical factors affecting cropping include site selection, organic and mineral fertilization, seed potato health, proper planting date, proper protection against weeds, pests and diseases. A factor that significantly reduces starch accumulation in tubers is excessive precipitation during the growing season [11].

Potatoes are considered as a plant with low previous crop requirements; however, a high level of its crop can be obtained only under conditions of appropriate soil culture. The introduction of cultivars resistant to cyst nematodes created a possibility of greater tolerance in the selection of previous crops for potatoes, but did not fully solve the threat resulting from the high share of potatoes in crop rotation. The number of cultivated plant species is limited, which in turn leads to simplification in crop rotation. The basic task of crop rotation is to make the most of the soil environment [12,13]. The crop rotation limits the impact of plants on the soil and the mutual influence of plants on each other and the soil on plants. According to the principle of rational rotation, the higher the crop yields, the less often it is grown in the same field [14,15]. Modern intensive and industrialized agriculture is striving for increasing production efficiency. In such conditions, most plants are grown in poor positions and often in monoculture. As a consequence, this leads to a decrease in yields, a decrease in soil fertility and its biological activity [16,17].

Among the agrotechnical factors determining potato yielding, the type of fertilization plays an important role. The use of fertilizers, both organic and inorganic, can significantly affect potato yield, but organic fertilizers and their extracts also improve soil fertility, its structure, and help plants combat pests and diseases [18]. Although the proper use of mineral fertilizers, especially nitrogen and phosphorus, is necessary to correct the imbalance of nutrients in infertile soils [19,20], it is well known that their use is not helpful in intensive agriculture, as it is often associated with reduced yield, soil acidity and nutrient imbalance.

Due to concerns related to soil depletion and nutritional imbalance resulting from the increased and massive use of NPS mineral fertilizers, which can be used to supplement soil nutrients, postulates research on the use of manure, which is an organic fertilizer [21]. However, the use of organic fertilizer alone may not fully satisfy the nutrient requirements of plants due to the low content and bioavailable nutrients, high application rates and ultimately the need to integrate with inorganic fertilizers [22,23,24]. On the other hand, the use of manure may be more important in the case of growing late potato varieties, as there are appropriate conditions for the uptake of nutrients during the period of intensive manure degradation in the soil [25].

The effect of fertilization on the growth and yield of potatoes was the subject of series of experiments [25,26,27,28]. The results to date indicate that mineral fertilizers have a more beneficial effect on the shape of potato crops than manure. Nitrogen is essential nutrient in potato production as the value of the other inputs cannot be fully realized unless N is applied to the crop in an optimum amount [29]. The literature data indicate that the application of a full dose of fertilizer during planting can lead to significant N losses, while the split use of N during tuber initiation can increase tuber swelling and thus increase tuber yield and quality [30,31]. However, excessive availability of N before tuber initiation leads to late season vegetative growth and delayed tuber swelling [32]. The quality of potato tubers is also a problem, because mineral fertilization can have a strong impact on its quality [33] and it has a significant impact on the chemical composition of potato tubers [34,35,36,37].

The significance of fertilizers in potato cultivation may increase simplified crop rotation, therefore, the aim of the study was to assess the yield and chemical composition of potato tubers grown in different previous crop systems depending on long-term varied organic and mineral fertilization. The most important advantage of the described study is conducting it in a long-term cycle. Both the assessment of cropping systems and different fertilization variants require longer studies due to the subsequent of their impact. Long research cycles make it possible to determine the sustainability of selected farming systems (cropping systems and fertilization).

## 2. Results and Discussion

Potato yield depends on many factors, both environmental and agrotechnical. Adequate hydration, soil quality or stress factors affecting the plant can significantly affect the yield [38,39,40]. The methods of fertilization and the method of breeding are also important. The average level of potato yielding in crop rotation, for the fertilization facilities included in the study (Table 1), was 17.4 t/ha for the 7-year research period, while in monoculture it was only 6.8 t/ha. A significant reduction (by 38%) of the potato yield grown in Poland in monoculture in earlier multi-year studies (1963–1991) was also observed by other authors [41]. The use of appropriate fertilization can significantly improve the potato yield [37]. The manure fertilization increased the yield in both cultivation systems to 32.4 and 15.5 t/ha, respectively, while the use of mineral fertilizers, in various combinations, did not lead to such a spectacular increase in the average yield. However, it was noticed that the combination of fertilization with manure and NPK addition had a positive effect on the yield. The reported yield was twice as high in the case of crop rotation and almost three times as high in the case of monoculture.

The positive effect of manure used combined with mineral fertilization on potato yielding was confirmed in many previous studies [42,43,44]. Beneficial effect of manure on the content of organic matter contributes to increasing water retention, reducing soil erosion and improving the physicochemical and biological properties of the soil [45]. However, in conventional farming systems, new yielding varieties cannot fully exploit the yield potential when fertilized with manure alone because their nutrient requirements are higher. The use of manure with NPK significantly increases the soil’s abundance of nutrients, leaving reserves of these nutrients in the soil for subsequent plants. The manure introduced into the soil also prevents the negative impact of nitrogen fertilizers on lowering the pH of the soil.

On the basis of the obtained results in the years used for research, a large variability of the yield level was noticed, from 19.6 to 31.6 t/ha in crop rotation and from 7.7 to 17.1 t/ha in monoculture (Table 2). The lowest crops were obtained in 2013 (for crop rotation) and in 2008 (for monoculture), although the least favorable weather conditions for potato growth were recorded in 2009. The negative impact of monoculture was the least marked in 2009, when the reduction in yield compared to rotation was 32.1%, and the greatest in 2008 (reduction in yield by 64.2%). In both systems of plant succession, the highest tuber yield (18.8–37.0 t/ha) was obtained after combined fertilization with NPK manure; the use of manure alone or only mineral fertilization resulted in lower yields in most of the analyzed years of experience.

Despite obtaining lower potato yields in self-cultivation (monoculture or short rotations), it results in profitable economic aspects. Taking into account producers’ income, potato cultivation may bring higher profits than other crops. However, longer crop rotations can increase soil productivity, maintain or even increase plant yield (profitability) and reduce the build-up of diseases, weeds and pests [46,47,48]. This will reduce the amount of fertilizers and pesticides used and thus lower costs in the long term [49,50,51].

Accumulation of nitrogen and, consequently, the yield of tubers are closely related to environmental factors, e.g., air temperature, water availability or soil properties [52]. No effect of the cultivation method on the average tuber dry matter content and starch content was observed, but the starch yield per hectare significantly increased, by 109%, when using crop rotation (Table 3). Although the excessive use of N fertilizer may cause leaf overgrowth and a decrease in tuber yield, its supply in sweet potato cultivation had a significant impact on the starch content in tubers [53]. Additionally, in the present study, it was shown that the application of fertilization with manure or NPK fertilization caused an increase in starch efficiency by 89.2% and 72.6%, respectively, but the combination of both fertilizers resulted in a higher starch efficiency than in the case of using both fertilizers separately. At the same time, many authors draws attention to the negative impact of high doses of nitrogen fertilization on the content of dry matter and starch in potato tubers [54,55].

Values within a column with same letter do not differ significantly. The most important yield response to nitrogen fertilization is the tuber size, which increases with increasing nitrogen dose. However, too intensive fertilization may cause the opposite effect. On the other hand, the number of tubers obtained may show a different response and is not always a good indicator of the effects of fertilization [56,57,58]. Additionally, a properly selected farming system can have a significant impact on the quality of the obtained potatoes [59,60]. Growing in monoculture resulted in a decrease in both the average weight of one tuber (57.7 vs. 73.2) and the number of tubers per plant (4.0 vs. 6.6), but also the tubers obtained were significantly smaller (69.5 vs. 83.3) than those obtained in the case of crop rotation (Table 4). The negative effect of growing potatoes in monoculture as an effect on the average number and weight of tubers per plant is also confirmed by the results of other authors [61], but the decrease in yield is noticed also for many other plants [62,63,64]. Reducing the average weight of tubers in a potato monoculture results in a lower commercial yield of tubers [50].

The use of the right fertilizer can contribute to better potato growth, but also easier tuber formation [65,66,67,68]. Simultaneous fertilization with manure and NPK had the best effect on the number and average weight of tubers. The applied manure can improved the soil quality [69,70], which may significantly affect the growth of plants and the setting of potato tubers. On the other hand, it ensures a long-lasting and even supply of plant nutrients, which can be used after the assimilation of compounds from mineral fertilizers [71].

Fertilization differentiated the content of the analyzed macroelements (N, P, K, Ca and Mg) in potato tubers to a greater extent than the crop rotation (Table 5). It was observed that the use of manure alone, and a mixture of manure and NPK, resulted in an increase in the content of the analyzed macroelements, but in the case of calcium, a much higher content was observed when only the NPK mineral fertilizer was used. As in the case of the size and number of tubers, this relationship is the result of a better supply of plants with nutrients from manure and their gradual availability during the growing season. The similar results of beneficial effect of organic fertilization in relation to mineral fertilization on the content of macroelements was also shown in the studies of other authors [72,73].

## 3. Materials and Methods

The research was conducted during the years 2007–2013 on the base of a long-term study established in 1958, at the Experimental Station Brody (52°26′ N; 16°18′ E), belonging to the Poznań University of Life Sciences (Poland). It was established as a randomized block design of four replicates on loam soil with the soil texture of sandy loam with underlying loams, bonitation class IIIb-IVa. The area of each plot was 55 m^2^. Potato was grown continuously since 1958 on the same plots. This is one of the oldest experiments in Poland, and one of the only three designed in a similar way in Europe, based on a valid statistical design. More details on the study area with information on soil fertility are provided in Blecharczyk et al. [74,75] and Szajdak et al. [76].

Varieties of potatoes resistant to cyst nematodes were grown in a 7-field rotation (potato—spring barley—alfalfa—alfalfa—spring oilseed rape—winter wheat—winter rye) and in monoculture. The presented study from the years 2007–2013 covers the next 8th rotation of the 7-field crop rotation (since 1958). With regard to continuous cultivation, this is the period between the 50th and 56th year of the potato monoculture. In 2007–2009, the ‘Satine’ potato variety was planted and in 2010–2013, the ‘Wineta’ variety. The experiment included 11 fertilization variants, of which the following 7 were included in the study: 1—control object without fertilization, 2—manure, 3—manure + NPK, 4—NPKCa, 5—NPK, 6—NP, 7—NK and 8—PK. Every year, mineral and organic fertilization was applied in the following doses per 1 ha: N—90 kg, P—26 kg, K—100 kg, manure—30 t and Ca—0.7 t. The chemical characteristics of the soil is determined after the potato harvest at the end of the research cycle in 2013 are presented in Table 6. The fungicidal, herbicidal, and insecticidal protection of plantations was conducted according to common recommendations on the Polish Institute of Plant Protection in Poznań. Weeds in potato plants in the years of research were controlled with the herbicide Sencor 70 WG (metribuzin) 0.3 kg/ha + Fusilade Forte 150 EC (fluazifop-P-butyl) 2.5 l/ha or Afalon 450 SC (linuron) 2.0 l/ha + Stomp 330 EC (pendimethalin) 4.0 l/ha, against fungal diseases, Penncozeb 80 WP (mancozeb) 3.0 kg/ha, Tanos 50 WG (cymoxanil + famoxate) 0.6 kg/ha or Bravo 500 SC (chlorothalonil) 3.0 l/ha were used alternately and for the control of Colorado potato beetle—Fastac 100 EC (alpha-cypermethrin) 0.1 l/ha, Karate Zeon 050 CS (lambda-cyhalothrin) 0.15 l/ha or Actara 25 WG (thiamethoxam) 80 g/ha.

After harvesting, the tuber yield and elements of its structure were determined on the basis of the number of tubers from 1 plant and the average weight of 1 tuber. Determination of dry matter content was conducted according to the AOAC method [77]. The starch content in tubers was determined by the Reimann Parow scale. In the dry weight of tubers, contents of total nitrogen (Kjeldahl method [78]), phosphorus and magnesium (colorimetrically [79,80]), potassium and calcium (atomic absorption spectrophotometry [81]) were determined.

In the years 2007–2013, the average temperatures in the period April—May were in the range 15.3–16.1 °C and were higher than in the years 1961–2006 (14.3 °C) (Table 7). The lowest amount of rainfall during potato growing was recorded in 2009 (327.4 mm), while the highest—in 2012 (550.8 mm). The least favorable distribution of rain occurred in 2009, in which the calculated Sielianinov’s hydrothermic coefficient was 1.1, which indicates the occurrence of a period of low humidity. This coefficient is calculated as the quotient of the monthly sum of precipitation and the sum of average daily air temperatures in a given month for the period in which the average daily temperature exceeds 10 °C [82,83]. The most favorable hydrothermal conditions for germination and growth of potatoes were observed in 2012, when Sielianinov’s coefficient value was 2.0, classifying this year of research as wet.

The results of the study were statistically evaluated using the analysis of variance (ANOVA). Significance of differences between objects was assessed by Tukey’s test for *α* = 0.05.

## 4. Conclusions

The use of different systems of cultivation and fertilization causes significant changes in the quantity and quality of the obtained crops. Potato cultivation in monoculture resulted in a significant reduction in tuber yield compared to crop rotation and a reduction in the number of tubers per plant and the average weight of one tuber. Manure fertilization, especially in combination with NPK mineral fertilizer, had a more favorable effect on the level of potato yielding and the content of N, P, K and Mg in tubers compared to only mineral fertilization, but decreased the content of dry matter, starch and calcium. Based on many years of field experience, it can be concluded that the optimal cultivation system is crop rotation combined with fertilization with organic and mineral fertilizers. Frequent cultivation of potatoes on the same field, used on specialist farms, becomes risky for economic and organizational reasons in the changing climate (rain shortage and high temperature).

## Figures and Tables

**Table 1:** Effect of cropping systems and fertilization on tuber yield of potato, mean of 2007–2013 (t/ha).

Fertilization	Crop Sequence		Mean
	Crop Rotation	Monoculture	
Control without fertilization	17.4 ef	6.8 i	12.1 F
Manure	32.4 b	15.5 fg	23.9 B
Manure + NPK	34.4 a	18.4 e	26.4 A
NPKCa	26.7 c	14.3 g	20.5 C
NPK	28.3 c	13.6 g	20.9 C
NP	19.0 e	8.4 i	13.7 E
NK	21.7 d	10.7 h	16.2 D
PK	21.7 d	10.7 h	16.2 D
Mean	25.2 A	12.3 B	-

Values marked with the same upper-case or lower-case letter do not differ significantly.

**Table 2:** Effect of cropping systems and fertilization on tuber yield of potato in years of research (t/ha).

Crop Sequence and Fertilization	Years						
	2007	2008	2009	2010	2011	2012	2013
Crop sequence							
Crop rotation	25.2 a	21.5 a	25.2 a	28.0 a	31.6 a	25.0 a	19.6 a
Monoculture	12.9 b	7.7 b	17.1 a	10.0 a	16.8 a	12.7 a	8.8 a
Fertilization							
Control without fertilization	13.4 e	11.4 de	13.6 e	11.1 d	14.4 d	12.2 c	8.7 d
Manure	22.4 b	19.0 a	24.8 ab	24.8 ab	33.7 a	25.1 ab	17.7 ab
Manure + NPK	26.7 a	18.8 a	27.5 a	27.7 a	37.0 a	27.9 a	19.1 a
NPKCa	18.7 c	15.1 bc	22.6 bc	22.7 b	26.2 b	23.3 b	14.9 bc
NPK	22.5 b	15.9 b	24.7 ab	20.8 b	26.1 b	21.6 b	15.0 bc
NP	15.6 de	10.9 e	17.9 d	13.8 cd	15.3 d	12.6 c	10.2 d
NK	17.3 cd	12.7 de	20.2 cd	15.6 c	19.5 c	14.6 c	13.3 c
PK	15.8 de	13.1 cd	18.2 d	15.9 c	21.3 c	13.8 c	14.9 bc
Years	19.1	14.6	21.2	19.0	24.2	18.9	14.2

Values within a column with same letter do not differ significantly.

**Table 3:** Content of dry matter and starch and yield of starch of potato tubers (mean of 2007–2013).

Crop Sequence and Fertilization	Dry Matter (%)	Starch (%)	Yield of Starch (t/ha)
Crop sequence			
Crop rotation	20.6 a	12.6 a	3.18 a
Monoculture	20.6 a	12.5 a	1.52 b
Fertilization			
Control without fertilization	20.8 ab	12.9 a	1.57 e
Manure	20.6 ab	12.4 a	2.97 ab
Manure + NPK	19.9 c	12.4 a	3.27 a
NPKCa	20.3 ab	12.6 a	2.56 c
NPK	21.0 a	13.0 a	2.71 bc
NP	21.1 a	12.7 a	1.75 de
NK	20.8 ab	12.4 a	1.99 d
PK	20.0 c	12.4 a	1.98 d

**Table 4:** Yield elements of potato tubers (mean of 2007–2013).

Crop Sequence and Fertilization	Weight of One Tuber (g)	No. of Tubers Per Plant	Percentage of Commercial Tubers (>4 cm) (%)
Crop sequence			
Crop rotation	73.2 a	6.6 a	83.3 a
Monoculture	57.7 b	4.0 b	69.5 b
Fertilization			
Control without fertilization	51.4 d	4.3 d	67.8 e
Manure	75.9 a	5.9 a	82.8 ab
Manure + NPK	81.4 a	6.1 a	85.8 a
NPKCa	70.6 b	5.6 ab	80.8 ab
NPK	70.0 b	5.6 ab	79.2 bc
NP	53.2 d	4.8 cd	67.3 e
NK	60.4 c	5.0 bc	72.2 de
PK	60.6 c	5.0 bc	75.1 cd

Values within a column with same letter do not differ significantly.

**Table 5:** Content of macronutrients in the dry matter of potato tubers, g/kg (mean of 2007–2013).

Treatments	N	P	K	Ca	Mg
Crop sequence					
Crop rotation	15.1 a	3.43 b	13.9 b	0.57 a	0.76 a
Monoculture	15.1 a	3.56 a	15.0 a	0.59 a	0.77 a
Fertilization					
Control without fertilization	14.5 bc	3.45 bc	14.1 bc	0.60 bc	0.75 c
Manure	15.7 ab	3.78 a	19.0 a	0.49 d	0.82 ab
Manure + NPK	16.7 a	3.86 a	18.5 a	0.48 d	0.84 a
NPKCa	14.7 bc	3.50 b	14.0 bc	0.64 a	0.76 bc
NPK	15.6 ab	3.31 c	11.9 de	0.63 ab	0.75 c
NP	14.9 bc	3.31 c	10.5 e	0.64 a	0.75 c
NK	14.5 bc	3.34 c	12.8 cd	0.59 bc	0.73 c
PK	14.2 c	3.42 bc	14.7 b	0.56 c	0.72 c

Values within a column with same letter do not differ significantly.

**Table 6:** Chemical properties of the soil (0–25 cm).

Fertilization	pH (1 M KCl)		C ((g/kg)		N ((g/kg)		P ((mg/kg)		K ((mg/kg)		Mg ((mg/kg)	
	cr	m	cr	m	cr	m	cr	m	cr	m	cr	m
Control without fertilization	5.46	5.75	5.88	5.44	0.61	0.50	68	97	36	50	35	32
Manure	6.01	6.17	10.76	8.62	1.10	0.88	145	163	201	177	74	56
Manure + NPK	5.98	6.23	11.40	9.66	1.17	1.02	182	177	246	267	66	69
NPKCa	6.82	6.87	8.43	7.44	0.73	0.78	121	135	103	104	40	43
NPK	5.66	5.54	6.72	6.55	0.64	0.64	99	120	84	83	21	25
NP	4.90	5.61	6.01	4.77	0.56	0.59	104	106	60	42	14	24
NK	5.40	5.35	6.55	6.66	0.56	0.56	63	109	120	82	14	23
PK	5.78	5.53	5.74	4.94	0.59	0.49	87	105	131	108	17	24

cr—crop rotation; m—monoculture.

**Table 7:** Weather conditions during potato vegetation since April to September.

Months	Years							Mean1961–2006
	2007	2008	2009	2010	2011	2012	2013	
Temperature (°C)								
IV	10.5	8.7	11.7	10.0	11.7	8.8	8.0	7.7
V	14.5	15.2	13.4	12.5	14.1	14.8	14.4	13.1
VI	19.2	19.1	15.7	18.7	18.6	16.0	17.3	16.4
VII	18.6	20.0	19.7	21.6	17.9	19.2	20.1	18.0
VIII	18.1	18.8	19.7	18.2	18.8	18.7	19.1	17.4
IX	13.2	13.9	15.6	12.4	15.3	14.3	12.9	13.3
Mean	15.7	16.0	16.0	15.6	16.1	15.3	15.3	14.3
Rainfall (mm)								
IV	4.8	120.7	13.3	38.9	13.9	22.9	15.4	37.5
V	149.8	19.5	85.3	92.7	34.0	77.2	69.8	54.8
VI	55.6	8.6	79.3	17.0	52.6	163.0	125.3	63.7
VII	96.2	80.1	68.1	98.2	175.4	197.6	67.3	76.8
VIII	70.9	171.5	31.4	109.6	34.5	60.1	51.5	65.1
IX	48.8	29.8	50.0	93.0	46.0	30.0	33.7	49.3
Sum	426.1	430.2	327.4	449.4	356.4	550.8	363.0	347.2
Sielianinov’s hydrothermic coefficient *								
IV	0.2	4.6	0.4	1.3	0.4	0.9	0.6	1.1
V	3.3	0.4	2.1	2.4	0.8	1.7	1.6	1.7
VI	1.0	0.2	1.7	0.3	0.9	3.4	2.4	1.3
VII	1.7	1.3	1.1	1.5	3.2	3.3	1.1	1.8
VIII	1.3	2.9	0.5	1.9	0.6	1.0	0.9	1.3
IX	1.2	0.7	1.1	2.5	1.0	0.7	0.9	1.1
Mean	1.5	1.5	1.1	1.6	1.2	2.0	1.3	1.4

* ≤0.5—drought; 0.6–1.0—semi-drought; 1.1–2.0—moist; >2.0—wet.

## Data Availability

All data generated or analyzed during this study are included in this published article.
